# Caustic soda ingestion in children under-5 years presenting for fluoroscopic examinations in an Academic Hospital in Ghana

**DOI:** 10.1186/s13104-015-1629-3

**Published:** 2015-11-17

**Authors:** Benard Ohene Botwe, Samuel Anim-Sampong, Benjamin Dabo Sarkodie, William K. Antwi, Jeannette Obeng-Nkansah, Gabriel G. N. A. Ashong

**Affiliations:** Department of Radiography, School of Biomedical and Allied Health Sciences, University of Ghana, P.O Box KB 143, Accra, Ghana; University of Ghana School of Medicine and Dentistry, Accra, Ghana

**Keywords:** Caustic soda, Children below five, Influencing factors

## Abstract

**Background:**

Disastrous effects and lifelong complications, ranging from respiratory and gastrointestinal burns to death can result from caustic soda ingestion. Accidental and non-accidental ingestions occur in different age groups. However, it is very troubling to find ingestion of caustic soda a very common occurrence among children below 5 years since they do not have the developmental level required to independently weigh up risks and are also under parental and societal protections. This study was therefore planned to investigate the ingestions of caustic soda by these children for purposes of proposing measures to curb the problem.

**Methods:**

Descriptive survey was employed for this study. A 14-item, semi-structure questionnaire was purposively issued to 57 parents/guardians whose wards had ingested caustic soda. Data was analysed with SPSS V.20.

**Results:**

Twenty-seven (47.4 %) children got access to the soda at storage, 1 (1.86 %) was administered accidentally by a sibling while 29 (50.9 %) ingested during soap preparation. In respect of the former, the majority got access because it was stored in soft drink and water bottles in their parents/guardians rooms or kitchen. For the later, the children got access to the left-over soda because the soap-makers failed to adhere to good storage and disposal practices.

**Conclusion:**

Storage of caustic soda in soft drink and water bottles in accessible places, and training of children to drink directly from bottles influence caustic soda ingestion in children under five. Non-compliance to good practices of storage and disposal of caustic soda during soap preparation increases exposure and access of children to caustic soda ingestion.

## Background

Ingestion of corrosive chemicals remains a common problem in the paediatric population and an important public health problem in many countries [1]. Generally, chemical or corrosive substances ingested by children are many. However, in the developing countries such as Ghana where traditional soap makers employed local soap making methods, the use of caustic soda, and ingestion of caustic soda by children is very common. Corrosive ingestion of caustic soda constitutes 0.3 % of paediatric admission in the Gambia [2] and 0.5 % in Nigeria [3], and was responsible for 0.8 % of total childhood mortality in the Gambia [4]. In Ghana, a sixfold increase in the number of children presenting with caustic soda ingestion to Okomfo Anokye hospital was noted between 2009 and 2010 [5].

This phenomenon is disturbing because potentially catastrophic presentation and lifelong complications result from caustic ingestion [6, 7]. Generally, caustic alkaline (soda) of pH >12 can induce damage [8] and ingestion leads to disruption of covalent bonds due to increased number of ions (OH). The hydroxide ion also interacts with collagen, muscle, leading to thrombosis of small blood vessels. Alkaline ingestion penetrates the layers of the oesophagus and other tissues that come into contact, resulting in increased permeability due to breakdown of epithelial barrier, and causing severe liquefactive necrosis, saponification of fats, emulsification of cellular membranes and solubilisation of proteins. These reactions result in burn injuries following ingestion of caustic soda. These injuries often involve the oral cavity, pharynx, larynx, oesophagus and stomach [6]. Stricture formation with inability to swallow food after the injury is inevitable in some cases, with 73 % of the children who sustain severe burn needing gastrostomy feeding [4]. For some victims the risk of perforations is also unavoidable [9] while long-term complications include increased lifetime risk of oesophageal carcinoma [10]. Evidence also suggests that approximately 80 % of caustic ingestions in the liquid phase occur in children younger than 5 years [11, 12] with those between 2 and 3 years being the commonest victims.

The degree and extent of these complications or severity of damage depends on several factors such as concentration of caustic substance, dose or quantity swallowed, fullness of the stomach and duration of contact with tissue or organs [4, 13] and the quality of care given at the initial management of the patient at presentation [14]. Solid and viscous phases produce more severe damage causing the management of corrosive ingestion and its sequel to constitute a medical challenge [8, 15].

In Ghana, currently, caustic ingestions are causing severe morbidity and mortality in children and represent a worsening problem. In 2014, the Daily Graphic [14] reported of 50 children who were on admission, in critical conditions, with some of them having their oesophagus completely damaged because of caustic soda ingestions. Besides, in 2013, out of the 36 children referred for fluoroscopic evaluation of the upper gastrointestinal (GI) tract at the Korle-Bu Teaching Hospital (KBTH) in Ghana, 38.9 % from the ages of 1–4 years reported with caustic soda ingestion. The age range of these patients makes it very disturbing because they do not have the developmental level required to weigh up risks independently and are supposed to be under parental and societal protections. A recent public debate in Ghana on how to curb this canker following a newspaper publication by the *Daily Heritage* with the heading; ‘Boy*, 3 dying slowly after drinking caustic soda… family appeals for help’* [16] has raised further questions on the factors associated with caustic soda ingestions in children under five. This study was therefore planned to investigate the factors influencing the ingestion of caustic soda by preschool children in order to propose measures to curb this problem.

## Methods

In accordance with ethical considerations, this study was approved by the Ethics and Protocol Review Committee of the School of Allied Health Sciences, University of Ghana. During the period of January 2014 to April 2015, 57 patients within the age range of 1–5 years with histories of caustic soda ingestion underwent upper GI fluoroscopic examinations at the KBTH, a tertiary referral centre in Ghana. Because most hospital facilities in Ghana do not have functional fluoroscopy equipment, most of the caustic soda victims needing fluoroscopy evaluation of the oesophagus are usually referred to KBTH. Parents and guardians were contacted and invited after the fluoroscopic study of their wards to participate in the study. They were assured of privacy, confidentiality and offered the opportunity to withdraw from the study at any point in time. After consenting to participate in the study, they were then purposively issued with a 14-item, self-designed, semi structure questionnaire (Appendix 2) which had been piloted successfully. Parents who could not read and write received interpretation assistance until all the questionnaires were completed. The questionnaires consisted of demographic section and factors that caused the children to ingest the caustic soda. The responses to the open-ended questions were grouped into themes determined by the responses, and later organised quantitatively together with the quantitative responses. Statistical package for social scientists (SPSS) version 20 was used to process the data. Descriptive statistics were then used to describe the findings.

## Results

Fifty-seven patients underwent fluoroscopy evaluation of their oesophagus subsequent to caustic soda ingestion. The accompanying parents and guardians victims agreed to participate and therefore a sample size of 57 respondents was used in this study.

### Demographic data

The majority (*n* = 32, 56.1 %) of the children who ingested caustic soda agents were 2–3 years old (Table 1). Their gender distribution is also shown in Table 1.

**Table 1:** Age and gender of the children who ingested caustic soda

Demographic variable	Number	Percent
Age (years)		
<2	1	1.8
2–3	32	56.1
>3–5	24	42.1
Gender		
Males	41	71.9
Females	16	28.1

The occupation of the patients’ parents/guardians and other demographics (age, level of education, number of children) are shown in Table 2.

**Table 2:** Respondents’ age, level of education, number of children and occupation

Demographic variable	Number	Percent
Age (years)		
≤ 24	16	28.1
25–35	22	38.6
36–45	7	12.2
≥46	12	21.1
Level of education		
No formal education	32	56.1
Hairdressing apprenticeship	12	21.1
Junior Secondary School (JSS)	10	17.5
Senior Secondary School (SSS)	3	5.3
Number of children		
≤3	19	33.3
4–5	29	50.9
6–7	9	15.8
Occupation		
Soap makers	41	72.0
Farmers	10	17.5
Traders	6	10.5

### Factors associated with caustic soda ingestion

According to the respondents 27 (47.4 %) of the children got access to the soda at storage, 29 (50.9 %) during soap preparation process while 1 (1.86 %) was administered accidentally by a 10 year old sibling. For the latter the caustic soda was stored in a drug bottle and placed at the same window with other medicines prescribed for the victim. Upon the father’s instruction to administer the prescribed drugs, the victim’s sibling could not differentiate the drugs and subsequently administered the caustic soda as well.

For respondents 27 (47.4 %) who indicated the children got access to the soda at storage, all of them said the soda was stored in soft drink and water bottles without proper fitting sealed covers, in their rooms or kitchen. For those who alleged their children accessed the caustic soda during soap preparation, 10 (34.5 %) confirmed the children got into contact with it when it was mixed with other materials and left in the open. A majority of 19 (65.5 %) respondents also said the children got into contact with the left-over soda and explained that soap makers preferred to finish the soap process before disposing of the left-over’s or waste.

In respect of where the ingestion happened, 29 (50.9 %) of the respondents alleged the incident occurred during soap preparation at home, while the other 28 (49.1) supposed that the incident happened when their wards were sent to soap making family relatives or neighbours, for up-keeping. They further indicated that their wards drank the caustic soda because they felt it was water or drink.

The majority 44 (77.2 %) of the respondents indicated they did not know much of the negative effects of caustic soda prior to their wards injury. Only 13 (22.8 %) of them admitted knowledge of the negative effects. All the respondents believed that most of the soap makers and other caustic soda users did not know much of the hazardous effects of caustic soda and hence made little efforts to properly secure it from children. All the parents/guardians also indicated that they trained their preschool children to drink directly from soft drink and water bottles.

## Discussion

Ingestion of caustic soda often used for soap making in the traditional homes can cause death, and children who survive it may stay deformed with varying lifelong complications [7]. This knowledge is lacking among parents or guardians who leave their wards with these local or household soap makers, whose activities usually expose children to caustic soda. Among the victims who ingested caustic soda, children aged between 2 and 3 years were most frequently injured (56.1 %), and the number of boys exceeded girls (71.9 vs. 28.1 %). These results are consistent with findings of Contini and colleagues [4] who also found boys to be affected more than girls.

Children mainly got access to caustic soda at storage due to poor storage precautions, lackadaisical attitudes, and non-compliance with good practices on the part of traditional soap makers. The corrosive substances used for household soap were often stored in soft drink and water bottles without proper sealed covers, and placed in rooms and kitchens where children played. Eventually when the children saw these bottles they assumed or misidentified them as containing soft drinks or water and drank from them irrespective of the contents. This scenario which was previously reported by Adedeji et al. [15] in Nigeria is further aggravated by the fact that the parents or guardians trained their preschool children to drink directly from soft drink and water bottles at home instead of cups as observed in this study. Weldon [5] has indicated that the increased incidence of children drinking caustic soda from bottles is due the introduction of bottled water in the Ghanaian environment. The old water bottles are re-used to store caustic soda which children drink mistaking it for water [5].

In other countries in different geographical locations, the incidence of corrosive oesophageal injuries has declined due to legislative efforts and stricter packaging standards [7, 17]. Unfortunately this may not be the situation in the developing countries [4]. Studies from South Africa and Nigeria reported that corrosive agent was readily available and within reach of the affected patients [13, 18]. This is because there are no restrictions to sale and handling of caustic chemicals in many developing countries including Ghana. Legislations to control the usage of caustic soda and ensure that containers are child-proof have been advocated for a long time [19]. It is important that they are enacted and enforced now. This is because the absence or ineffective regulation of potentially harmful chemical agents makes the environment unsafe and constitutes substantial risk to both children and adults [4].

Moreover, it is apparent that 50.9 % of caustic soda ingestions occur during soap preparation process due to poor handling of left-over caustic soda. Soap makers often forget and do not put left-over caustic soda into hiding but always preferred to finish the soap process before. As a result, preschool children get access to these corrosive substances. The fact that the caustic substances are mixed with other substances and left in the open without proper safety measures also influences children access to and risk of ingestion.

Only 1 out of 57 children was accidentally administered with the corrosive substance by a relative. The event to this action per the study is due to poor storage coupled with irresponsible parenting. The questions to ask are: How can caustic soda, a corrosive substance, be stored in a bottle and be placed at the same window where children drugs are also kept? And why should a parent instruct a 10 year old child to administer drugs to a preschool child? Obviously this mix-up where the caustic soda is accidentally administered is bound to happen.

Children are to be protected by their parents or guardians and the society at all times. Therefore, it may suggest that these children with these kinds of accidental caustic ingestions are more likely to live in ‘unsafe homes or communities’ with regard to accessibility to toxic agents.

In our study, the majority of children with caustic soda ingestions were in families whose parents did not have formal education, ply local soap making, undertake petty trading and farming as occupation. Unfortunately, the high costs of hospital admission, surgery, laboratory and radiologic examinations, coupled with the high cost of transportation to the referral centre for those living farther away are usually beyond the reach of many of these individuals resulting in late presentation of the injuries and further complications [20]. Fluoroscopy evaluation of the oesophagus for instance, in these victims often involves the use of water-soluble contrast agents which are very expensive and as such result in deferral of such diagnostic examinations.

Some researchers have also observed that repeated hospital visits also results in loss of work for parents and neglect of other children at home [11]. Since many of these respondents have four or more number of children, their children may suffer unduly for some of these irresponsible activities that have resulted in the caustic soda ingestions.

Effective legislation on caustic soda usage, educating parent/guardians on corrosive substances and further protecting children by the state are important measures that can be used to curb the onset of caustic soda ingestion in children below 5 years.

### Conclusion

Poor storage precautions where caustic soda substances are stored in soft drink and water bottles in accessible places and lackadaisical attitudes on the part of traditional soap makers, poor handling of left-over caustic soda during soap making and accidental administration of the corrosive substances influenced caustic soda ingestion in children below 5 years. Training of children to drink directly from bottles also influences caustic soda ingestion in children under 5 years. Corrosive ingestion is a considerable burden especially among people with little or no formal education and poor socio-economic status. This group of people should be targeted for education about creating safe working environments. Educating parents and soap makers will increase awareness of dangers associated with caustic soda and other corrosive substance ingestions. This is needful to guard against indiscriminate handing of these corrosive agents. Enforcement of chemical hazards legislation to control the usage of caustic soda is required to ensure that containers are child-proof to prevent easy access to corrosive agents by children.

A suggested algorithm for preventing caustic soda ingestion in children under 5 years is presented in Appendix 1.

### Limitations

The study used relatively small sample size. Also, the study focused on a referral population to a tertiary hospital, so it did not include children with minor ingestions that were managed locally without referral.

## Abbreviations

pH: potential of hydrogen
OH: hydroxyl ions
GI: gastrointestinal
KBTH: Korle-Bu Teaching Hospital
